# Perceived fussy eating in Australian children at 14 months of age and subsequent use of maternal feeding practices at 2 years

**DOI:** 10.1186/s12966-017-0582-z

**Published:** 2017-09-11

**Authors:** Rebecca Byrne, Elena Jansen, Lynne Daniels

**Affiliations:** 1 Centre for Children’s Health Research, School of Exercise and Nutrition Sciences, Queensland University of Technology, 62 Graham St, South Brisbane, QLD 4101 Australia; 2 School of Exercise and Nutrition Sciences, Queensland University of Technology, Kelvin Grove, QLD 4069 Australia

**Keywords:** Dietary intake, Food refusal, Fussy eating, Maternal feeding practices, Maternal perception, Obesity, Overweight, Picky eating, Structural equation modelling, Toddlers

## Abstract

**Background:**

Concerns about fussy eating are common amongst parents of young children. However, studies of the long-term impact of fussy eating show mixed results with regard to adequacy of dietary intake and child growth. This may be in part because there is no accepted definition of fussy eating and studies measure the construct in different ways, commonly relying on parent perception. This longitudinal analysis explores maternal and child characteristics associated with maternal perception of her toddler as a fussy eater in early toddlerhood and subsequent use of feeding practices at 2 years.

**Methods:**

Mothers completed a self-administered questionnaire at child age 14 months, describing perception of their child as fussy/not fussy and child behaviour. Intake was assessed using a single 24-h recall and weight was measured by research staff. At child age 2 years mothers completed the validated 28-item Feeding Practices and Structure Questionnaire (FPSQ-28).

Weight-for-age z-score (WAZ) was derived from WHO standards. Gram daily intake of fruit, vegetables and meat/alternative and a dietary diversity score were determined. Maternal/child characteristics independently associated (*p* ≤ 0.05) with perception of child as a fussy eater were determined using logistic regression. Variables were combined in a structural equation model assessing the longitudinal relationship between child/maternal characteristics, perception of child as a fussy eater and eight FPSQ factors.

**Results:**

Mothers’ (*n* = 330) perception of her child as a fussy eater at age 14 months, was associated with higher frequency of food refusal and lower WAZ (*R* ^2^ = 0.41) but not dietary intake. Maternal perception as fussy (age 14 months) was associated with four FPSQ factors at 2 years (*n* = 279) - Reward for Eating, Reward for Behaviour, Persuasive Feeding and Overt Restriction, *x* ^*2*^/df = 1.42, TLI = 0.95, CFI = 0.95, RMSEA = 0.04(0.03–0.05), PCLOSE = 0.99.

**Conclusions:**

Lower relative child weight and food refusal prompted mothers to perceive their child as fussy. These behaviours in healthy weight children most likely reflect self-regulation of energy intake and neophobia. This perception was prospectively associated with use of non-responsive feeding practices, which may increase obesity risk. Future interventions could directly address perceptions of growth and fussiness, supporting parents to understand food refusal as developmentally appropriate behaviour in healthy young children.

**Trial registration:**

ACTRN12608000056392. Registered 29 January 2008.

**Electronic supplementary material:**

The online version of this article (10.1186/s12966-017-0582-z) contains supplementary material, which is available to authorized users.

## Background

Anecdotally, parents have a strong desire to raise a child who is a ‘good eater’ and children are routinely praised for ‘eating all the food on their plate’ [1]. Concerns about fussy eating and underweight are common amongst parents in developed countries [2, 3] with fussy (or picky) eating reported to occur in 25–40% of toddlers [3]. This is in stark contrast to public health priorities which overwhelmingly relate to consumption of too much food and prevalence of childhood obesity [4]. The relationship between fussy eating and child outcomes, such as adequacy of dietary intake and growth trajectory is unclear [5–10]. This is perhaps because the construct of fussy eating is measured in a myriad of different ways [11].

From a theoretical perspective, fussy eating is defined as the rejection of a substantial amount of familiar and unfamiliar foods, potentially resulting in limited dietary variety and food intake [12, 13] However, studies rarely use objective measure of dietary intake (most likely because of the complexity and labour-intensive nature of this type of data collection) and instead rely on parent report/perception of fussiness [3, 10, 14, 15]. However, it is important to explicitly examine the criteria that parents use to label their child as a fussy eater. Parents, health professionals and researchers may not necessarily define fussy eating in the same way [16] and hence interpret study-specific questions such as ‘is your child a fussy/picky eater’ [5, 7] quite differently. Perception may also differ between mothers, fathers and non-parental carers. Children could be labelled as fussy because they do not consume the type and/or amount of *food* perceived as appropriate [17] and parents may differ in the type, frequency and extent of *behaviours* they consider to be problematic. The Food Fussiness (FF) scale of the Child Eating Behaviour Questionnaire (CEBQ) [18] is frequently used to characterise fussiness according to child behaviour, with five items related to food refusal, tasting new foods and enjoying variety. In a cross-sectional analysis of 3-year-old twin children (*n* = 1330 pairs) from the Gemini cohort in the United Kingdom, FF was inversely correlated with liking of both fruit and vegetables [19] but we are unaware of any examination of the relationship with food intake amongst toddlers.

Our underlying premise is that a mother pieces together information (consciously or not) about her child’s behaviour, physical appearance and amount/type of dietary intake, which inform her perception and her subsequent feeding practices. Non-responsive feeding practices are actions which a parent employs during feeding which may interfere with a child’s ability to recognise and attend to internal hunger and satiety cues and increase obesity risk [20, 21]. Examples of these practices include pressuring a child to eat more than they want to, and using food as a reward to encourage eating or to encourage desired behaviour. In another analysis of the Gemini cohort, this time of when the twins were 16-month-old (*n* = 2026) [22], between-family analyses indicated that ‘pressure to eat’ and ‘instrumental feeding’ i.e. using food to encourage healthy food consumption/good behaviour were positively associated with Food Fussiness on CEBQ [23]. An analysis of a subset of 274 Gemini twin pairs that were most discordant on food fussiness, found that mothers used more ‘pressure to eat’ and ‘instrumental feeding’ with the twin perceived as fussier. Similarly a cross-sectional survey of 413 parents of Australian children aged 1–10 years found that ‘persuasive feeding’ and ‘reward for eating’ (constructs which correspond to ‘pressure to eat’ and ‘instrumental feeding’ respectively) were both positively associated with Food Fussiness on CEBQ [23]. Overall, there is evidence that parent perception of fussiness is, at least cross-sectionally, associated with non-responsive feeding practices which may in turn be counterproductive in our obesogenic environment and contribute to obesity risk.

The aims of this analysis were to 1. Identify what maternal and child characteristics – demographics, behaviour, measured dietary intake - were associated with maternal perception of her child as a fussy eater in early toddlerhood (12–16 months), and 2. Explore the prospective relationship between child/maternal characteristics and maternal perception of child as a fussy eater at 12–16 months, with maternal feeding practices at age 2 years.

## Methods

### Design and participants

This is a secondary analysis of data from mother-child dyads who participated in the control group of NOURISH, a randomised controlled trial that evaluated an intervention promoting positive feeding practices in very young children [24], and an additional sample of mothers recruited for the South Australian Infants Dietary Intake (SAIDI) Study [25]. SAIDI participants were recruited simultaneously and using the same protocol as NOURISH. Recruitment has been described in detail [24, 26]. Briefly, a consecutive sample of mothers (aged ≥18 years) delivering healthy term infants (≥ 37 weeks gestation and birthweight ≥2500 g) at maternity hospitals in Brisbane, Queensland (*n* = 3) and metropolitan (*n* = 5) and regional (*n* = 7) South Australia were approached within 72 h post-delivery. Eligible mothers were asked to provide consent to be contacted when infants were 4- to 7-months-of-age, for full enrolment in the study. Approval was gained from a total of 11 human research ethics committees including Queensland University of Technology and Flinders University (QUT HREC 00171 Protocol 0700000752).

### Measurements

Maternal and child demographic data were collected during initial recruitment in hospital including gender, maternal age at birth of child (years), and maternal university education.

Participants attended study-specific outcome assessment clinics when children were 12–16 months of age (June 2009 to June 2010). Child weight was measured by trained staff using a standard protocol. Weight was measured twice, lying or sitting on electronic infant scales with no nappy or clothes (except a singlet), to the nearest 10 g. A third measurement was taken if the first two differed by more than 50 g. In regional areas, participants were measured at their local child health or general practitioner clinic. Mothers completed a self-administered questionnaire containing five questions regarding fussy eating and child behaviour (Table 1).

**Table 1:** Items on self-administered questionnaire at child age 12–16 months used to characterise maternal perception of fussy eating and child behaviour

Question	Response	Dichotomised variable used in regression model
Maternal perception of her child as a fussy eater:		
Do you think your child is a picky or fussy eater?	*Very picky, Somewhat picky, Not picky, Not sure*	Fussy (*very picky*, *somewhat picky* combined), Not fussy (*not picky*, *not sure* combined)
Child behaviour**:**		
How often does your child refuse food?	*Very often, Often, Sometimes, Hardly ever*	Often (*Very often, Often*), Not often (*Sometimes, Hardly ever)*
Does your child ever refuse food they usually eat?	*Hardly ever, Yes*	
How willing is your child to eat unfamiliar foods?	*Very willing, Willing, Neutral, Unwilling, Very unwilling*	Willing (*Very willing, Willing*), Not willing (*Neutral, Unwilling, Very unwilling*)
Who decides how much food your child eats – you or your child?	*You only, Mostly you, You and your child equally, Mostly your child, Your child only*	Mother (*You only, Mostly you, You and your child equally*), Child (*Mostly your child, Your child only*)

Within two weeks of this assessment, child dietary intake was assessed via telephone by a dietitian using a single three-pass 24-h recall [27]. Recalls were collected on week days and weekends. The mother was asked to recall everything her child ate or drank in the previous 24 h, starting from midnight on the previous day, with quantities estimated using household measures (metric cup, tablespoon and teaspoon). A visual aide designed to improve estimation, which showed actual size illustrations of these measures, was provided at assessment. Items from the recall were entered into FoodWorks Professional version 9 using the AUSNUT 2007 database from the 2007 Australian National Children’s Nutrition and Physical Activity Survey [28]. An additional database containing commercial infant products was created by study staff. Mixed dishes prepared at home were added to FoodWorks as a recipe or if there were ≤three ingredients, entered into FoodWorks as separate items. A study-wide data checking protocol meant that any children with very high or very low estimates of energy intake - <3000kj or >6000kj had their FoodWorks file checked against the original recall, to correct any possible errors. Food recall data were exported from FoodWorks into an Access database and merged with an eight digit food group code which allows identification of each unique food [29]. Study staff allocated additional eight digit codes to infant foods and mixed dishes/recipes with codes allocated based on the predominant ingredient. A comprehensive analysis of dietary intake data for this cohort has been published elsewhere [27].

When the child was aged 2 years, mother-child dyads attended a second assessment at which time mothers completed another questionnaire containing 28 items of the Feeding Practices and Structure Questionnaire (FPSQ-28) [30] and an item regarding Family Meal Setting (Additional file 1).

### Derived variables

Weight-for-age z-scores were derived from measured weight using WHO standards [30] and all data was exported into SPSS 22.0 for analysis.

#### Maternal perception of child as a fussy eater at 12–16 months and child behaviour

Responses to the question ‘Do you think your child is a picky or fussy eater?’ were dichotomised to form the variable ‘perception of child as a fussy eater’: *fussy* / *not fussy* as per the methodology of Carruth et al. [5] (Table 1). Table 1 also shows the four questionnaire items chosen to characterise child behaviour regarding refusal of familiar and unfamiliar food.

#### Dietary intake

Intake (grams/day) of three food groups were derived based on their unique eight digit food group code – i. fruit (excluding 100% fruit juice), ii. vegetables (including beans and lentils) and iii. Meat/alternatives (including fish, poultry, and eggs). Australian and international studies indicate that dairy and cereal groups contribute the highest proportion of daily energy intake amongst young children with these food groups tending to be consumed in excess of dietary recommendations [27, 31, 32]. The most recent representative study of Australian children aged 2–3 years indicated 95% consumed dairy and 97% cereals, with each food group contributing 21% and 27% of daily estimated energy intake, respectively [31]. Therefore they were not considered an ‘at risk’ food groups requiring further investigation in this analysis.

A dietary diversity score was calculated for each child i.e. number food groups consumed on 24-h recall with a potential score of 0–9 [33] (vitamin A-rich fruits and vegetables [34], other fruit; other vegetables; legumes and nuts; meat, poultry and fish; breads, cereals, roots and tubers; eggs; dairy/alternatives; fats and oils). Australia’s second dietary guideline states that by 12 months of age children should “*enjoy a wide variety of nutritious foods… each day*” [35].

### Analyses

Bivariate analyses were conducted using independent t-test or Mann-Whitney test for continuous variables and Pearson’s chi-squared test for categorical variables to determine differences between children perceived as *fussy* or *not fussy* for thirteen independent variables that described maternal and child demographics; child food refusal and dietary intake. Variables with a significant bivariate association (*p* ≤ 0.05) with perceived fussiness were entered into a logistic regression model to determine characteristics independently associated with perception of child as a fussy eater (dependant variable), with results expressed as odds ratios and 95% CI. Using Mplus (v7.4), these variables were combined in a structural equation model with maternal perception of child as a fussy eater and 28 items of the FPSQ-28 [30] and the additional single item measuring family meal setting (Additional file 1). The weighted least squares estimator (WLSMV) was used to determine standardised regression weights for the pathways in the model since items were treated as ordinal categorical variables [36]. Model fit was determined using normed chi-square (*x* ^2^/df), Tucker-Lewis index (TLI), comparative fit index (CFI) and root mean square error of approximation (RMSEA) with 90% CI and probability (PCLOSE).

## Results

Characteristics of mother-child dyads completing an assessment at age 12–16 months (*n* = 330) are shown in Table 2. Notably, no children were underweight [30].

**Table 2:** Characteristics of mothers and toddlers (*N* = 330)

Characteristic	Mean (SD)	n (%)
Child age (months)	13.8 (1.3)	
Birthweight (kg)	3.5 (0.4)	
Weight-for-age z-score^a^ at 12–16 months of age	0.58 (0.86) Range: −1.6 to 2.9	
Male gender		165 (49)
Maternal age at birth of child (years)	30.3 (5.0)	
Maternal university education		193 (58)
Family income^b^ ≥ 70,001 $AUD		199 (60)
Child Dietary intake	Median (IQR)	
Fruit intake (g) on 24-h recall^c^	118 (60–192)	
Vegetable intake (g) on 24-h recall^c^	80 (21–152)	
Meat/alternatives intake (g) on 24-h recall^c^	49 (15–108)	
Dietary diversity score^d^	6 (5–7)	

^a^Weight-for-age z-score calculated using WHO Anthro (2008); 92% weight measured vs 8% self-reported based on measure with GP/nurse in rural areas

^b^n = 321; Median Australian gross income, 2008 - all household types = 67,000 $AUD [56]

^c^ Intake on single 24-h recall of whole sample; Fruit: fresh, canned, dried, cooked, infant food or mixed dish where fruit is the predominant ingredient; Vegetables: fresh, canned, cooked, beans and lentils, infant food or mixed dish where vegetable is the predominant ingredient; Meat/alternatives: fish, poultry, beef, lamb, pork, game meats, egg, nuts and seeds, infant food or mixed dish where meat/alternative is the predominant ingredient

^d^ Diversity score from 0 to 9 representing number of different food groups (vitamin A-rich fruits and vegetables; other fruit; other vegetables; legumes and nuts; meat, poultry and fish; breads, cereals, roots and tubers; eggs; dairy/alternatives; fats and oils) consumed on 24-h recall

Thirty-one percent of mothers defined their child as a fussy eater - not fussy, *n* = 232; fussy, *n* = 98 (‘very picky’ *n* = 10 and ‘somewhat picky’ *n* = 88 combined). Bivariate analyses comparing children perceived as ‘fussy’ versus ‘not fussy’ revealed group differences for nine (highlighted by *italics*) of the 13 variables considered: maternal age, *maternal education*, *child age*, gender, *WAZ, fruit intake, vegetable intake*, meat/alt intake; diversity; *child decides amount of food eaten; how willing is your child to eat unfamiliar foods?; how often does your child refuse food?*; and *does your child ever refuse food they usually eat?* (additional file 2). However, only five variables remained significantly associated with maternal perception in the adjusted cross sectional analysis (Table 3): weight-for-age z-score and the four measures of child behaviour. Mothers’ perception of her child as a fussy eater was associated with higher frequency of refusal of familiar and unfamiliar food, the child choosing amount eaten and lower WAZ; but not intake of fruit, vegetables, meat or dietary diversity; x^2^(9) = 109.36, *p* < 0.001, −2 Log likelihood = 287.56, R^2^ = 0.41(Nagelkerke).

**Table 3:** Variables independently associated with maternal perception of child as a fussy eater (N = 330)

Independent variables	Dependant variable: perception of child as a fussy eater (not fussy^a^, n = 232; fussy, n = 98); OR (95% CI)
Child weight-for-age z-score	0.69 (0.48–0.99)*
Child age (months)	1.17 (0.92–1.48)
Maternal university education; yes, *n* = 191	1.58 (0.85–2.96)
Fruit intake (g) on 24-h recall^b^	0.99 (0.99–1.00)
Vegetable intake (g) on 24-h recall^b^	0.99 (0.99–1.00)
Who decides amount of food eaten; child, *n* = 144	1.94 (1.07–3.51)*
How willing is your child to eat unfamiliar foods?; unwilling, *n* = 76	4.52 (2.33–8.75)***
How often does your child refuse food?; often, *n* = 47	6.12 (2.62–14.30)***
Does your child ever refuse food they usually eat?; yes, *n* = 158	2.31 (1.23–4.34)*

*p ≤ 0.05; ***p ≤ 0.001, *x* ^2^(9) = 109.36, *p* < 0.001, −2 Log likelihood = 287.56, 0.41 (Nagelkerke)

Mean(sd) child age 13.8 (1.3) months; 49% male; WAZ using WHO standards [55]

^a^Referent group

^b^Intake on single 24-h recall of whole sample; Fruit: fresh, canned, dried, cooked, infant food or mixed dish where fruit is the predominant ingredient; Vegetables: fresh, canned, cooked, beans and lentils, infant food or mixed dish where vegetable is the predominant ingredient

**Fig. 1:**
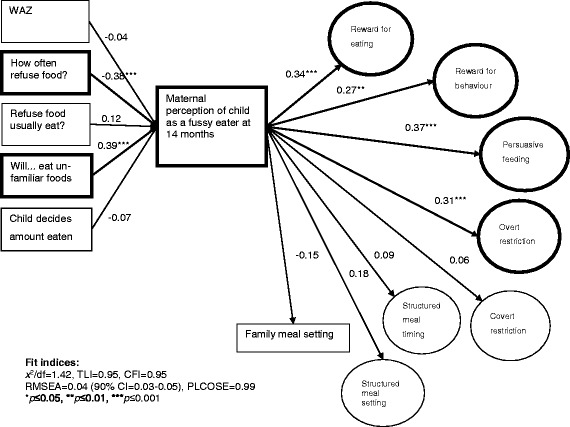
Relationships between child characteristics and maternal perception of child as a fussy eater at 14 months, and use of feeding practices at 2 years (*n* = 279). For ease of reading figure, error terms associated with variables or measurement component of FPSQ-28 are not shown. WAZ: Weight-for-age z-score using WHO Standards [55]. How often does your child refuse food? Very often, often, sometimes, hardly ever. Does your child ever refuse food they usually eat? Hardly ever, yes. How willing is your child to eat unfamiliar foods? Very willing, willing, neutral, unwilling, very unwilling. Child decides amount of food eaten. Mother only, mostly mother, mother and child equally, mostly child, child only

Longitudinal data was available for 279 mother-child dyads. The model showing the relationship between child variables, maternal perception and the FPSQ-28 is shown in Fig. 1 and was a good fit, *x* ^*2*^/df = 1.42, TLI = 0.95, CFI = 0.95, RMSEA = 0.04 (0.03–0.05), PLCOSE = 0.99. Maternal perception of her child as a fussy eater at age 12–16 months was directly associated with four factors of the FPSQ-28 at 2 years - Reward for Eating (β = 0.34, *p* < 0.001), Reward for Behaviour (β = 0.27, *p* < 0.01), Persuasive Feeding (β = 0.37, *p* < 0.001), and Overt Restriction (β = 0.31, *p* < 0.001). The relationships between WAZ, child decides amount of food eaten, or does your child ever refuse food they usually eat and maternal perception were no longer significant.

## Discussion

In everyday lexicon young children tend to be categorised as either a ‘good’ eater or a ‘fussy’ eater. Amongst this sample of healthy children, a third were perceived as fussy and this perception was associated with maternal feeding practices that may be counterproductive in our obesogenic environment.

The finding that approximately 30% of mothers perceived their child as a fussy eater is consistent with published prevalence data [5, 37], and the analysis identified factors that explain 40% of the variance in maternal perception. However, instead of aligning with the theoretical definition of fussy eating which is largely focussed on limited intake - there was no difference in the amount of fruit, vegetables or meat/alternatives eaten, or dietary diversity, between those children perceived as fussy and those that were not - perception was related to child behaviour not food intake. This supports previous qualitative analysis indicating “*parents believe that picky eating is not only defined by the food the child eats, but also by the child's overall behaviours and attitudes toward mealtimes*” [38].

The relevant behaviours - child refused food often or unwilling to eat unfamiliar foods – do support the definition of fussy eating as the rejection of both novel and familiar foods [12]. However, these behaviours could also be indicative of normal toddler development i.e. self-regulation of energy intake (refusal of familiar foods), neophobia (refusal of unfamiliar foods) and emerging autonomy [39]. Rate of growth in toddlers slows in comparison to infancy [40] with a relative decline in energy requirements, which may result in refusal of familiar foods in response to intrinsic cues of satiety. Food neophobia, the rejection of foods that are novel or unfamiliar, also increases markedly at this age [12]. This heritable trait [41, 42] is thought to play a protective function, discouraging children from ingesting toxic substances as they become more mobile [43]. The age group 12–16 months coincides with the development of autonomy and independence [39] and meal times are perhaps one of the few areas that toddlers can exert their growing autonomy – by refusing to eat when they are not hungry. It is unknown whether mothers distinguish between (and respond differently to) refusal of familiar versus unfamiliar foods.

Kerzner et al. [44], in their ‘Pyramidal Representation of Young Children’s Feeding Behaviors’ identify four child behaviour categories – normal, misperceived feeding problems, milder feeding difficulties and feeding disorders. Their paper urges clinicians to take parent concerns seriously, even if misperceived, given the potential for parents in response to these concerns to “*adopt inappropriate feeding practices*”. This is consistent with our findings. We postulated that a mother pieces together information about her toddler which informs (consciously or unconsciously) her perception and subsequent feeding responses/practices. In our study, the child who refused familiar and unfamiliar food more frequently was perceived as a fussy eater at age 12–16 months, which was associated with use of non-responsive maternal feeding practices at 2 years. These included specific practices such as using favourite foods in exchange for good behaviour or offering children food when they are upset (Reward for behaviour), and insisting children eat food despite not being hungry or showing disapproval when a child does not eat (Persuasive feeding). All of these practices may prompt a child to eat for reasons other than hunger and may interfere with self-regulatory ability [43, 45, 46]. In a longitudinal study of 222 Australian children, food fussiness at age two years (measured within a ‘food approach’ construct using items from the CEBQ) was correlated with use of instrumental feeding practices i.e. use of food as a reward, a year later [47]. Our findings also support the suggestion of Walton et al. that labelling a child as fussy may contribute to sub-optimal feeding interactions and endorse their call to reconceptualise fussy/picky eating i.e. resistance during eating can be considered children’s agency in communicating eating preferences instead of deviant behaviour [48].

The finding that maternal perception of fussy eating was not associated with objectively assessed dietary intake in the NOURISH/SAIDI sample highlights the importance of accurately assessing the child variables informing maternal perception. Perception of fussiness defined according to actual inadequate dietary intake, might prompt use of different feeding practices than those identified in this analysis. This research could also be extended by considering whether particular feeding practices have differential outcomes. For example is persuasive feeding associated with adverse growth trajectory? Does offering preferred foods effectively reduce exposure to a wide range of foods, resulting in a narrow range of food preferences in later life [49, 50]? Understanding different ‘types’ of fussy eating, differential feeding practices employed in response to these, and subsequent effects on a range of child eating behaviour, dietary intake and growth outcomes may facilitate the development of targeted interventions to address the unique needs of parent-child dyads at each stage in this continuum. Use of non-responsive practices could be reduced by explicitly designing interventions to assist mothers (and other carers) to understand developmentally appropriate eating behaviour.

Limitations of the current analysis include not measuring and adjusting for feeding practices used at 12–16 months. When NOURISH and SAIDI were designed, there were no tools validated to measure feeding practices at both 12–16 months and 2 years. The FPSQ-28 is validated for use at 2 years, but not yet at younger ages. The relationship between dietary variety and maternal perception was not assessed. While use of a single 24-h recall is valid for group level estimates of food intake [51] and a simple measure of diversity [33, 52], it is not suitable to assess micronutrient intake. It is also not appropriate to assess dietary variety, which may also inform a mother’s perception of her child as fussy. Also, the premise that child behaviour informs maternal perception is limited by using maternal report of child behaviour. If a mother is concerned or frustrated about a child refusing food, then it is possible she could systematically overestimate the frequency of this behaviour [53]. This bias would strengthen the positive statistical relationship between frequency of food refusal and perception of fussiness, but highlights that a mother’s perception of her child may be more influential in determining what she does, than the child’s actual intake or behaviour. The analysis may have been strengthened by comparing variables of interest across children perceived as ‘very picky’ versus ‘somewhat picky’ versus ‘not picky’ however given only 3% (*n* = 10) of the total sample were characterised as ‘very picky’ there is inadequate power to investigate differences between these groups. The analyses reported here do not enable consideration of important questions related to potential prospective impact of maternal feeding practices on child weight outcomes and hence the hypothesised role of feeding practices as a mediator of reported associations between fussy eating and child weight. During an additional analysis that added child WAZ at 2 years to the model, fit became unacceptable (data not shown) and hence no conclusions could be drawn.

The sample comprised first-time mothers who were slightly older with a higher level of education compared to the Australian average [54] and hence generalisability is unclear. It is unknown whether the prevalence of perceived fussy eating differs according to socioeconomic status (SES) and it is feasible that the characteristics which mothers use to inform their perception vary according to SES. The contribution of fathers to feeding and family life generally is acknowledged, but was outside the scope of this study.

Strength of this study lies in the use of SEM to simultaneously assess the relationship between child characteristics, maternal perception and maternal feeding practices. Many studies rely on parent report only, to investigate the relationship between the ‘fussy child’ and maternal practice. However, this analysis was able to take into account multiple factors mothers used to inform their perception, delineating between refusal of familiar and unfamiliar foods and objective measures of dietary intake and weight.

## Conclusion

Overall, there was no difference in food intake between children perceived as fussy versus not. Mothers appear to be interpreting developmentally appropriate feeding behaviour as fussiness in the leaner but healthy weight child. Importantly, this perception of typical child eating behaviour as fussiness was prospectively associated with use of non-responsive feeding practices, which may teach children to eat in response to cues other than hunger or satiety, disrupting self-regulation of energy intake and increasing obesity risk. Interventions to modify feeding practices should support parents and clinicians to interpret food refusal as normal behaviour in healthy young children and directly address perceptions of healthy child growth and developmentally appropriate behaviour.

## Additional files

Additional file 1: Factors and corresponding items of the Feeding Practices and Structure Questionnaire (FPSQ-28) with an additional item measuring family meal setting. (DOCX 16 kb)

Additional file 2: Results of bivariate analysis comparing children perceived as ‘fussy’ versus ‘not fussy’. (DOCX 15 kb)

## Abbreviations

FPSQ: Feeding Practices and Structure Questionnaire,
SEM: Structural Equation Modelling
WAZ: weight-for-age z-score
WHO: World Health Organisation
