# Supplementary material for: Perceived fussy eating in Australian children at 14 months of age and subsequent use of maternal feeding practices at 2 years
Source: Int J Behav Nutr Phys Act. 2017 Sep 11;14:123. doi: 10.1186/s12966-017-0582-z (PMC5594597; doi:10.1186/s12966-017-0582-z)
Supplement: Supplementary file 1 — Factors and corresponding items of the Feeding Practices and Structure Questionnaire (FPSQ-28) with an additional item measuring family meal setting. (DOCX 16 kb) [file 12966_2017_582_MOESM1_ESM.docx]

Additional file 1. Factors and corresponding items of the Feeding Practices and Structure Questionnaire (FPSQ-28) with an additional item measuring family meal setting

| **Factor** | **Item (s)** |
| --- | --- |
| Reward for Eating | - ...do you encourage the child to eat something by using food as a reward (for example, “If you finish your vegetables, you will get some fruit)? - When your child refuses food they usually eat, do you encourage to eat by offering a food reward (e.g. dessert)? - I use desserts as a bribe to get my child to eat his/her main course - ...do you warn the child that you will take a food away if the child doesn’t eat (for example, “If you don’t finish your vegetables, you won’t get fruit”)? |
| Reward for behaviour | - I offer my child his/her favourite foods in exchange for good behaviour - In order to get my child to behave him/herself I promise him/her something to eat - I reward my child with something to eat when (s)he is well behaved - I give my child something to eat to make him/her feel better when (s)he is feeling upset |
| Persuasive feeding | - If my child says “I’m not hungry” I try to get him/her to eat anyway - When your child refuses food they usually eat, do you insist your child eats it? - I praise my child if (s)he eats what I give him/her - ...do you reason with the child to get him/her to eat (for example, “Milk is good for your health because it will make you strong”)? - ...do you tell the child to eat something on the plate (for example, “Eat your beans”)? - ...do you say something to show your disapproval of the child for not eating? |
| Overt restriction | - I have to be sure that my child does not eat too many sweet foods (lollies, ice-cream, cake or pastries) - I have to be sure that my child does not eat too much of his/her favourite foods - I intentionally keep some foods out of my child’s reach - If I did not guide or regulate my child’s eating, (s)he would eat too many junk foods |
| Covert restriction | - How often do you avoid going with your child to cafes or restaurants which sell unhealthy foods? - How often do you avoid buying lollies and snacks eg. potato chips and bringing them into the house? - How often do you not buy foods that you would like because you do not want your children to have them? - How often do you avoid buying biscuits and cakes and bringing them into the house? |
| Structured meal timing | - I allow my child to wander around during a meal - I insist my child eats meals at the table - How often are you firm about where your child should eat? |
| Structured meal setting | - I let my child decide when (s)he would like to have her meal - I decide when it is time for my child to have a snack - I decide the times when my child eats his/her meals |
| Family meal setting | - My child eats the same meals as the rest of the family |
