# Supplementary material for: Perceived fussy eating in Australian children at 14 months of age and subsequent use of maternal feeding practices at 2 years
Source: Int J Behav Nutr Phys Act. 2017 Sep 11;14:123. doi: 10.1186/s12966-017-0582-z (PMC5594597; doi:10.1186/s12966-017-0582-z)
Supplement: Supplementary file 2 — Results of bivariate analysis comparing children perceived as ‘fussy’ versus ‘not fussy’. (DOCX 15 kb) [file 12966_2017_582_MOESM2_ESM.docx]

Additional file 2: Results of bivariate analysis comparing children perceived as ‘fussy’ versus ‘not fussy’

| **Variable** | **Fussy  (n=98)** | **Not fussy (n=232)** | ***p***^a^ |
| --- | --- | --- | --- |
| Maternal age (years); mean (sd) | 31.0 (5.0) | 30.1 (5.0) | 0.12 |
| Maternal university education, %(n); yes, n=191 | 67 (66) | 54 (125) | 0.03 |
| Child age (months); mean (sd) | 14.1 (0.13) | 13.7 (0.08) | 0.004 |
| Child gender, %(n); male | 46 (45) | 52 (120) | 0.39 |
| Child WAZ; mean (sd) | 0.42 (0.08) | 0.66 (0.06) | 0.02 |
| Fruit intake (g)^b^; median (IQR) | 105 (44-163) | 125 (67-202) | 0.04 |
| Vegetable intake (g)^b^; median (IQR) | 51 (9-130) | 92 (27-241) | 0.01 |
| Meat/alternatives intake (g) ^b^; median (IQR) | 36 (9-108) | 50 (19-108) | 0.28 |
| Dietary diversity^c^; mean (sd) | 5.5 (1.3) | 5.7 (1.4) | 0.28 |
| Child decides amount of food eaten, % (n); yes, n=144 | 58 (57) | 38 (87) | <0.001 |
| How willing is your child to eat unfamiliar foods? % (n); unwilling, n=76 | 48 (47) | 13 (29) | <0.001 |
| How often does your child refuse food? % (n); often, n=47 | 37 (36) | 5 (11) | <0.001 |
| Does your child ever refuse food they usually eat? % (n); yes, n=158 | 74 (72) | 37 (86) | <0.001 |

^a^ using independent t-test or Mann-Whitney test for continuous variables, depending on distribution; and Pearson’s chi-squared test for categorical variables
^b^ Intake on single 24-hour recall of whole sample; Fruit: fresh, canned, dried, cooked, infant food or mixed dish where fruit is the predominant ingredient; Vegetables: fresh, canned, cooked, beans and lentils, infant food or mixed dish where vegetable is the predominant ingredient; Meat/alternatives: fish, poultry, beef, lamb, pork, game meats, egg, nuts and seeds, infant food or mixed dish where meat/alternative is the predominant ingredient
^c^ Potential score of 0-9. Food groups included: vitamin A-rich fruits and vegetables; other fruit; other vegetables; legumes and nuts; meat, poultry and fish; breads, cereals, roots and tubers; eggs; dairy/alternatives; fats and oils.
